# The role of genetic liability for psychiatric disorders and personality traits in post covid syndrome: data from three Nordic population cohorts

**DOI:** 10.1016/j.eclinm.2026.103928

**Published:** 2026-05-07

**Authors:** Liam Quinn, Ida Henriette Caspersen, Ingibjörg Magnúsdóttir, Yue Wang, Mischa Lundberg, Jesper Gådin, Kadri Kõiv, Anna Bára Unnarsdóttir, Arna Hauksdóttir, Bitten Aagaard, Bjarke Feenstra, Christian Erikstrup, Christina Mikkelsen, DBDS Genetic Consortium, Jakob Hjorth von Stemann, Nanna Brøns, Josephine Gladov, Lotte Hindhede, Maiken Astrup Madsen, Lea Arregui Nordahl Christoffersen, Jacob Træholt, Bertram Kjerulff, Jens Kjærgaard Boldsen, Johan Skov Bundgaard, Line Hjorth Stjernholm Nielsen, Mette Skou Bentsen, Khoa Manh Dinh, Joseph Dowsett, Maria Didriksen, Michael Schwinn, Lisette kogelman, Anne Grosen, Tanya Techlo, Christina MIkkelsen, Thomas F. Hansen, Susan Mikkelsen, Kathrine A. Kaspersen, Laura Barrett Ryø, Rasmus Tanderup Jensen, Casia Nursyifa, Caroline Thue Hvilsom, Emil Jørsboe, Dorte Helenius, Edda Bjork Thordardottir, Elzabeth C. Corfield, Erik Sørensen, Frank Geller, Jakob Thaning Bay, Jóhanna Jakobsdóttir, Johanne Hagen Pettersen, Kelli Lehto, Khoa Manh Dinh, Kristjana H. Ásbjörnsdóttir, Lill Trogstad, Maria Didriksen, Mie Topholm Bruun, Ole Andreassen, Per Magnus, Ragnhild Eek Brandlistuen, Sisse Rye Ostrowski, Søren Brunak, Thomas Werge, Thor Aspelund, Helga Ask, Unnur Anna Valdimarsdóttir, Ole Birger Vesterager Pedersen, Lea Arregui Nordahl Christoffersen

**Affiliations:** a Department of Clinical Immunology, Zealand University Hospital, Køge, Denmark; b Centre for Fertility and Health, Norwegian Institute of Public Health, Oslo, Norway; c Centre of Public Health Sciences, Faculty of Medicine, University of Iceland, Reykjavik, Iceland; d Institute of Biological Psychiatry, Mental Health Services, Copenhagen University Hospital, Copenhagen, Denmark; e Estonian Genome Centre, Institute of Genomics, University of Tartu, Estonia; f Department of Clinical Immunology, Aalborg University Hospital, Aalborg, Denmark; g Department of Congenital Disorders, Statens Serum Institut, Copenhagen, Denmark; h Department of Clinical Immunology, Aarhus University Hospital, Aarhus, Denmark; i Department of Clinical Medicine, Aarhus University, Aarhus, Denmark; j Department of Clinical Immunology, Copenhagen University Hospital, Rigshospitalet, Copenhagen, Denmark; k Novo Nordisk Foundation Center for Basic Metabolic Research, Faculty of Health and Medical Science, Copenhagen University, Copenhagen, Denmark; l Population Health Sciences, Bristol Medical School, University of Bristol, Bristol, UK; m Bristol Medical School, University of Bristol, Bristol, UK; n Department of Method Development and Analysis, Norwegian Institute of Public Health, Oslo, Norway; o Department of Neuroscience, Faculty of Health and Medical Sciences, University of Copenhagen, Copenhagen, Denmark; p Department of Clinical Immunology, Odense University Hospital, Odense, Denmark; q Centre for Precision Psychiatry, Institute of Clinical Medicine, University of Oslo, Oslo, Norway; r Division of Mental Health and Addiction, Oslo University Hospital, Oslo, Norway; s Department of Child Health and Development, Norwegian Institute of Public Health, Oslo, Norway; t Department of Psychology, University of Oslo, Oslo, Norway; u Department of Clinical Medicine, Faculty of Health and Medical Sciences, University of Copenhagen, Copenhagen, Denmark; v Novo Nordisk Foundation Center for Protein Research, University of Copenhagen, Copenhagen, Denmark; w Icelandic Heart Association, Kopavogur, Iceland; x PsychGen Center for Genetic Epidemiology and Mental Health, Norwegian Institute of Public Health, Oslo, Norway; y Department of Medical Epidemiology and Biostatistics, Karolinska Institutet, Stockholm, Sweden; z Department of Epidemiology, Harvard T.H. Chan School of Public Health, Boston, MA, USA

**Keywords:** Post covid syndrome, Psychiatric disorders, Genetic scores, Risk profiles

## Abstract

**Background:**

Post-COVID syndrome (PCS) remains a substantial public health concern, yet its genetic determinants are poorly understood. Psychiatric disorders and related traits influence infection risk and acute COVID-19 outcomes, raising the possibility that shared genetic liability may also shape long-term symptom persistence. We examined whether polygenic scores (PGS) for schizophrenia (SCZ), bipolar disorder (BPD), major depressive disorder (MDD), attention-deficit/hyperactivity disorder (ADHD), and neuroticism are associated with PCS, and explored potential pathways underlying these associations.

**Methods:**

We analysed three population-based cohorts from Denmark, Norway and Iceland (total n = 80,726; PCS cases = 6103) between March 2020 and June 2022. PCS was defined as COVID-19–related symptoms lasting ≥3 months. Logistic regression models estimated associations between standardised PGS and PCS among COVID-19 positive individuals, adjusting for ancestry principal components. Additional analyses assessed associations with COVID-19 infection. In a subset with available personality data, models were additionally adjusted for measured Neuroticism (NEO-FFI). Supplementary analyses examined associations between PGS and COVID-19 infection risk, and we conducted LD score regression (LDSC) and proteomic analyses as contextual genetic and biological characterisations of the PGS traits.

**Findings:**

Higher PGS for neuroticism, MDD and ADHD were consistently associated with increased odds of PCS across all cohorts (ORs per SD: ∼1.07–1.16). Quintile analyses showed a graded pattern, with the highest PGS quintile displaying 30–45% higher odds of PCS than the lowest. PGS for SCZ and BPD showed no evidence of association with PCS. PGS associations with COVID-19 infection were weaker and inconsistent. In the subset with available personality data, these associations remained essentially unchanged after adjusting for measured Neuroticism, indicating that they are not solely attributable to observed personality differences. LDSC and proteomic analyses did not alter the primary interpretation of the PGS–PCS associations.

**Interpretation:**

Polygenic liability for neuroticism, MDD, and ADHD is associated with increased risk of PCS across three national cohorts. This pattern is consistent with shared symptom-related liability contributing to these associations, although the data do not allow differentiation between post-viral sequelae and pre-existing symptom liability. While PGS explain only a modest proportion of PCS variance, they provide useful insight into underlying psychiatric and personality-related factors associated with persistent symptom reporting following COVID-19 infection.

**Funding:**

The study was funded by EU Horizon REACT study (101057129), environMENTAL study (101057429), Nordforsk (project numbers 105668 and 138929), and the Independent Research Fund Denmark (0214-00127B).

**Research in context**

**Evidence before this study**

Post-COVID syndrome (PCS) affects millions worldwide, yet its biological determinants remain unclear. Psychiatric disorders and related traits are known to influence susceptibility to infection and acute COVID-19 severity, and polygenic scores (PGS) have emerged as tools to quantify genetic liability for these traits. However, a search of PubMed on June 5, 2025 using the terms “long COVID” OR “post-COVID syndrome” combined with “polygenic score,” “genetic risk,” or “psychiatric genetics” identified no studies examining whether psychiatric or personality-related PGS are associated specifically with PCS. Existing genetic studies have focused largely on acute COVID-19 outcomes, leaving a gap in knowledge regarding the contribution of psychiatric genetic liability to PCS.

**Added value of this study**

In three national population-based cohorts from Denmark, Norway and Iceland (n = 80,726; PCS cases = 6103), we demonstrate that genetic liability for neuroticism, major depressive disorder (MDD) and attention-deficit/hyperactivity disorder (ADHD) is consistently associated with increased risk of PCS. These associations were replicated across cohorts with differing demographics and selection mechanisms and remained significant after correction for multiple testing. In a subsample with available NEO-FFI data, the associations for neuroticism-PGS, MDD-PGS, and ADHD-PGS persisted after adjusting for observed neuroticism, suggesting that the genetic associations are not solely attributable to measured personality traits. Additional analyses, including linkage disequilibrium score regression and inflammatory protein associations, provided contextual information about the broader genetic and biological profiles of these psychiatric traits.

**Implications of all the available evidence**

Together, these findings provide evidence that genetic liability to specific psychiatric and personality-related traits, particularly those characterised by affective, cognitive, and somatic symptom tendencies contribute to the likelihood of PCS. The selective pattern of associations suggests that symptom-related liability may play an important role in how persistent post-COVID symptoms are experienced or reported, although the present data cannot distinguish post-viral effects from persistence or amplification of pre-existing symptom liability. While PGS are not clinically predictive at the individual level, identifying patterns of genetic liability may help guide future research into psychiatric, behavioural, and symptom-related factors shaping PCS, particularly in studies with detailed pre-infection phenotyping and longitudinal symptom assessment.

## Introduction

Post-COVID syndrome (PCS) often referred to as ‘long COVID’ is characterised by symptoms persisting for at least three months after SARS-CoV-2 infection.^1^^,^^2^ Neurological and neuropsychiatric complaints including fatigue, memory problems, concentration difficulties and insomnia are among the most common features.^3^ Symptom severity and persistence amongst PCS individuals can vary significantly including those with severely impaired quality of life.^4^ Although PCS is now well described clinically,^5^ its underlying mechanisms remain unclear.^6^ Proposed pathways include persistent immune dysregulation,^7^ autonomic and endothelial disturbances,^8^ and psychological and behavioural responses to infection.^9^ Given the marked heterogeneity of PCS, it is likely that multiple biological and behavioural processes jointly shape long-term outcomes.

Psychiatric disorders have long been associated with increased susceptibility to infection and more severe disease courses across multiple diseases including infectious respiratory diseases.^10^ Recent genetic studies extend this to COVID-19, showing that polygenic liability to several major psychiatric traits are associated with SARS-CoV-2 infection and acute COVID-19 severity.^9^^,^^11^ These associations are typically interpreted through two broad mechanistic pathways. First, many psychiatric traits share immune and stress-related biological pathways that may influence antiviral responses and degree of inflammation.^12^^,^^13^ Second, psychiatric traits are linked to behavioural and lifestyle factors including physical inactivity, smoking, risk-taking and delayed help-seeking.^14^^,^^15^ These lifestyle factors may result in increased exposure to infection, worsened acute disease, and an influence post-acute recovery.^15^, ^16^, ^17^ Both mechanisms suggest a plausible link between psychiatric genetic liability and PCS risk, even though PCS-specific genetic studies are almost entirely lacking.

Polygenic scores (PGS) provide a means of quantifying individual genetic liability for complex traits and assessing their association with PCS.^18^^,^^19^ We examined PGS for schizophrenia (SCZ), bipolar disorder (BD), major depressive disorder (MDD), attention-deficit/hyperactivity disorder (ADHD), and the personality trait neuroticism. These traits were selected because they represent major psychiatric domains that have been linked in prior research to infection risk, symptom reporting, and health-related behaviour. MDD, ADHD, and neuroticism include affective, cognitive, and somatic symptoms such as fatigue, reduced concentration, and negative affectivity that overlap with common PCS symptom domains.^20^, ^21^, ^22^, ^23^ SCZ and BD were included for contrast, as they involve symptom profiles that are clinically distinct from PCS, yet are also associated with well-described immunological and inflammatory alterations in the psychiatric literature.^24^^,^^25^ Including both symptom-overlap traits and immunologically distinct conditions allowed us to evaluate whether associations with PCS generalise across psychiatric domains or are selective to traits that share particular symptom or immunological features with PCS.

In this study we examined whether polygenic liability to SCZ, BPD, MDD, ADHD and neuroticism is associated with PCS in three population-based cohorts from Denmark, Norway and Iceland, and explored potential explanations linking psychiatric genetic risk to long-term post-infectious outcomes.

## Methods

### Study population

#### Denmark

The Danish study sample was based on participants in the Danish Blood Donor Study (DBDS) with available genotype information and PCS status assessed from at least one of three questionnaires distributed between December 2020 and May 2022 through the password protected governmental email system, e-boks.^26^ DBDS is a nationwide ongoing prospective cohort study, recruiting blood donors from all Danish regions. DBDS is described in further detail elsewhere including ethical procedures, data collection and laboratory handling of materials.^27^^,^^28^

In DBDS, DNA from whole blood was genotyped at deCODE Genetics, using the Illumina Global Screening Array chips and long range phased using Eagle2 [6]. QC was performed prior to imputation and included removal of sample duplicates, genotype rates lower than 0.98, and ancestry outliers (less than 90) and EIGENSOFT (v 6.0.1) [8]. Only variants with a genotype rate higher than 0.95, a MAF >0.01, and a p-value <10ˆ(−6) for HWE were retained for downstream imputation. In addition, LD pruning using a window size of 100 markers shifting by 25 markers, removed half of every variant-pair with a genotypic rˆ2 > 0.1. The LD pruned markers were used to calculate heterozygosity, as well as identity by state and sex. Finally, samples with outlying heterozygosity (>5 SD from the median), a sample from each pair of samples with an IBS >0.9, samples where reported sex did not match sex determined by genotype, and all A/T and C/G markers were removed. Imputation of non-genotyped SNPs was then performed at deCODE genetics, using an inhouse workflow [9], where graphtyper [1] formed the reference panel. The reference panel at deCODE Genetics, Iceland included a total of approximately 25,000 individuals of North-Western European ancestry, with around 8500 Danish individuals sequenced as part of other studies.

#### Norway

The Norwegian Mother, Father and Child Cohort Study (MoBa) is a population-based pregnancy cohort study conducted by the Norwegian Institute of Public Health.^29^ Participants were recruited from all over Norway during 1999–2008. The women consented to participation in 41% of the pregnancies. The cohort includes around 95,000 mothers, 75,000 fathers and 114,000 children. Genotype data was procured from blood samples taken during pregnancy.^30^ Genotype calling, quality control, phasing, and imputation were carried out using the MoBaPsychGen pipeline, developed by Corfield et al.^31^ Between March 2020 and June 2022, active adult participants (mothers and fathers) were invited to answer electronic questionnaires on the COVID-19 pandemic every 14–30 days. In June 2022, 60,780 participants answered a questionnaire which included self-reported symptoms related to PCS (response rate 44%). For this study, we used a subset of MoBa mothers and fathers with available genotype and questionnaire data on PCS symptoms (n = 45,125).

#### Iceland

The Icelandic study sample was from the Icelandic COVID-19 national resilience cohort (C-19 Resilience), which was established in April 2020 and eligible for all Icelandic speaking individuals aged 18 years or older in Iceland.^32^ A total of 23,960 individuals were recruited at baseline (April 2020–May 2021), and three waves of follow-ups were completed by February 2022. Participants were invited to complete a series of web-based questionnaires and provide information on demographics, lifestyle and general health, as well as COVID-19 status at each assessment. Genetic data was obtained through linkage with the deCODE genetics database, which has collected blood samples for more than 160,000 Icelanders by August 2017.^33^

For further details regarding processing of genetic data in all three cohorts see Supplementary methods.

### Quality control procedures

Genetic quality control (QC) procedures were applied in all three cohorts to ensure ancestry homogeneity and to address relatedness. Individuals were restricted to those of European ancestry, defined through principal component analysis (PCA) or clustering with European reference panels, and outliers were excluded. Relatedness was assessed using kinship coefficients, with one individual removed per related pair prioritising retention of cases. For Iceland, where genetic homogeneity is high, population structure was accounted for by including genetic principal components as covariates and applying genomic correction factors to adjust for relatedness. Full details of ancestry filtering, relatedness handling, and QC thresholds are provided in the Supplementary Methods.

### PGS calculation

Polygenic scores (PGSs) were constructed in three cohorts (DBDS, MoBa, and C-19 Resilience) using genome-wide association study (GWAS) summary statistics obtained from publicly available sources or directly from study authors (Supplementary Table S1). In all cohorts, GWAS summary statistics underwent preprocessing and quality control, after which polygenic scores were generated using Bayesian shrinkage methods with ancestry-matched linkage disequilibrium reference panels. Cohort-specific details of genotype quality control, summary statistic cleaning, and PGS computation pipelines are provided in the Supplementary Methods Table S1.

### Measures

#### Covid-19 phenotypes

In all cohorts, individuals were classified as COVID-19 positive if they had a register confirmed or self-reported positive test for COVID-19. Individuals with PCS were identified through self reported COVID-19-related symptoms persisting for three months or more.

#### Pre-pandemic psychiatric disorders

##### Denmark

Severe pre-pandemic (before 01 January 2020) psychiatric disorders were defined as a hospital based diagnosis of one of the following disorders according to the international classification of diseases the 10th revision (ICD-10): any psychiatric disorder: all F prefixed diagnosis codes, schizophrenia:F20, bipolar disorder: F30–F31, major depressive disorder: F32–F33, and attention deficit hyperactivity disorder (ADHD): F90.0. Information on psychiatric disorders was obtained from the national patient register, holding information on 95–100% of psychiatric disorders diagnosed at a psychiatric hospital department since 1995.^34^

##### Norway

Pre-pandemic (before 01 January 2020) psychiatric diagnoses were identified using ICD-10 codes from chapter F (mental and behavioural disorders) recorded in the Norwegian Patient Registry. The registry contains nationwide data on specialist health care use, including hospital-based psychiatric diagnoses.^35^

##### Iceland

Information on participants' history of psychiatric disorders was collected at baseline using the question ‘During your lifetime, has a physician or psychologist ever diagnosed you with a mental disorder? (No; Yes [depression, bipolar disorder, panic attacks, generalized anxiety disorder, adjustment disorder, post-traumatic stress disorder, schizophrenia or other psychotic disorder, substance use disorder, sleep disorder, social phobia, obsessive compulsive disorder, attention deficit hyperactivity disorder (ADHD), or other])’.

### Statistical analyses

Characteristics of the study population(s) were assessed with count and percentages including a breakdown of year of birth, sex and prevalence of pre-pandemic psychiatric diagnoses in each of the cohorts according to covid-19 status.

In our primary analysis, we examined the associations of the five PGSs with PCS, using all COVID-19 positive individuals without PCS as controls. This was done by performing logistic regression modeling in R using the glm package and the family = “binomial” argument. Results are presented as odds ratios (ORs) with 95% confidence intervals (CIs). The first ten principal components derived from the narrow ancestry analysis (see above) were used as covariates in all PGS based logistic regression models to control for potential impacts of population stratification. Subsequently, to ascertain the distribution of genetic risk for statistically associated PGS with PCS we performed a further logistic regression analysis converting the PGS's into factor variables comprising five levels representing quintile ranking of the individual PGS. The second to fifth quintiles of PGS were each compared to the first quintile which was used as a reference group.

We additionally performed logistic regression analysis for our five PGS and the outcome of COVID-19 infection, using all uninfected individuals as controls. For these five analyses the first 10 principal components were used as covariates to control for potential population stratification.

In the Danish cohort, a subsample of participants completed the NEO Five-Factor Inventory (NEO-FFI). To evaluate whether measured Neuroticism accounted for the observed polygenic associations with PCS, we repeated the logistic regression analyses in this subsample and included the continuous NEO-FFI Neuroticism score as an additional covariate. Models were otherwise specified identically to the main PGS analyses, adjusting for age, sex, and the first ten ancestry principal components. Results are reported as odds ratios (ORs) per SD increase in PGS with corresponding 95% confidence intervals.

Finally, to check for possible interaction effects with our PGS's and other variables in relation to PCS as an outcome, we performed additional logistic regression analyses adding interaction terms of each PGS with year of birth, sex and pre-pandemic psychiatric diagnoses, respectively.

### LDSC

We estimated pairwise genetic correlations between psychiatric traits, influenza infection, and severe COVID-19 using linkage disequilibrium score regression (LDSC).^36^ For the psychiatric traits (schizophrenia, bipolar disorder, major depressive disorder, neuroticism, and Attencion deficit hyperactivity disorder), we used the same GWAS summary statistics as in the main polygenic risk score (PRS) analyses. Summary statistics for influenza infection were obtained from the recent large-scale GWAS by Burren et al.,^37^ and summary statistics for severe COVID-19 were taken from the extended European GWAS meta-analysis conducted by Degenhardt et al.^38^

All datasets were harmonised to the GRCh38 reference genome and reformatted to LDSC specifications. Genetic correlations were computed using the ldsc_regression_rG workflow (version 1.2.2) executed within a Singularity container, applying baselineLD v2.2 annotations, LD weights, and a predefined list of excluded genomic regions.

### MESOSCALE protein marker analysis

Plasma protein concentrations were obtained using the MESO Scale Discovery (MSD) electrochemiluminescence platform as described in the Danish blood donor proteomics study.^39^ In brief, multiplex electrochemiluminescence immunoassays were used to quantify 36 inflammatory and immune-related proteins across multiple panels, including cytokines, chemokines, and endothelial activation markers. All sample processing, assay calibration, and quality control procedures followed those described in the original study. For the present analysis, we used the normalized concentration values provided in the cleaned dataset: calc_conc_imp_norm (imputed, normalized concentrations) as the primary outcome measure, and calc_conc_norm (non-imputed normalized concentrations) for sensitivity analyses. We examined the association between each PGS and each protein marker using a series of linear regression models including age, sex and the first ten principal components as covariates. We calculated both global FDR (across all tests) and assay-specific FDR corrections on the p-values obtained from regression models. Additionally, for the three proteins that showed robust global FDR–significant associations (ICAM-1, CCL22, and IL-1RA), we performed a top–bottom 5% contrast by defining groups based on the ADHD PRS distribution (≤5th vs. ≥95th percentile), refitting the regression models with a binary PGS-group indicator to obtain percent differences with 95% confidence intervals.

All statistical analyses in this study were performed in the R programming environment with a significance level of 0.05. All reported p-values from the main analyses were adjusted for multiple testing using the false discovery rate (FDR) procedure.

### Ethics statement

For DBDS participants, oral and written informed consent was obtained from all participants. The study was approved by The Scientific Ethical Committee of Central Denmark (M-20090237). Additionally, the biobank and research database have been approved by the Danish Data Protection Agency (2007-58-0015).

MoBa is regulated by the Norwegian Health Registry Act. Written informed consent was obtained from all participants. The current study was approved by The Regional Committees for Medical and Health Research Ethics (14140).

All participants in the Icelandic C19-Resilience cohort signed an electronic informed consent before answering a web-based questionnaire. The study was approved by the National Bioethics Committee (NBC 20-073, 21-071) as well as the National Data Protection Authority.

### Role of the funding source

The funders of the study had no role in study design, data collection, data analysis, data interpretation, or writing of the report. The funder of the study had no role in study design, data collection, data analysis, data interpretation, or writing of the report.

## Results

In the three cohorts there were a total of 80,726 participants including 6103 individuals with PCS (Table 1). Across all three cohorts, individuals with PCS were more often female and younger than both COVID-19 cases without PCS and non-COVID-19 individuals. For example, females comprised 75.3% of PCS cases in Norway (vs. 66.2% of COVID-19 cases without PCS), 59.5% in Denmark (vs. 53.6%), and 75.9% in Iceland (vs. 68.9%). PCS cases also tended to come from younger birth cohorts; in Norway, 68.6% of PCS individuals were born in the 1970s, with older groups under-represented, a pattern mirrored in Denmark and Iceland. Pre-pandemic psychiatric diagnoses were more common among PCS individuals in Norway (22.9% vs. 14.2% in COVID-19 without PCS) and Iceland (36.3% vs. 24.9%), while numbers were too low in Denmark (9.3% among PCS) to assess clear trends (Table 1).

**Table 1:** Descriptive characteristics of the study sample.

Nation	Denmark (DBDS)			Norway (MoBa)			Iceland (C-19 resilience)		
Phenotype	PCS	COVID-19	Non-covid	PCS	COVID-19	Non-covid	PCS	COVID-19	Non-covid
Number of individuals									
n	1036	13,350	7373	4002	12,568	28,555	1063	5969	7012
Sex									
Male	420 (40.5%)	6198 (46.4%)	3636 (49.3%)	990 (24.7%)	4251 (33.8%)	10,331 (36.2%)	256 (24.1%)	1858 (31.1%)	2137 (30.5%)
Female	616 (59.5%)	7152 (53.6%)	3737 (50.7%)	3012 (75.3%)	8317 (66.2%)	18,224 (63.8%)	807 (75.9%)	4111 (68.9%)	4875 (69.5%)
Year of birth									
Before 1950	25 (2.4%)	600 (4.5%)	721 (9.8%)	<5	<5	27 (0.1%)	106 (9.8%)	981 (16.4%)	1484 (21.2%)
1950s	193 (18.6%)	2656 (19.9%)	2369 (32.1%)	22 (0.5%)	100 (0.8%)	427 (1.5%)	289 (27.2%)	1708 (28.6%)	2133 (30.4%)
1960s	330 (31.9%)	3532 (26.5%)	2166 (29.4%)	814 (20.3%)	2717 (21.6%)	9126 (31.2%)	347 (32.6%)	1618 (27.1%)	1681 (24.0%)
1970s	261 (25.2%)	2955 (22.1%)	1133 (15.4%)	2741 (68.6%)	8623 (68.6%)	17,441 (61.2%)	219 (20.6%)	1003 (16.8%)	1021 (14.6%)
1980s	144 (13.9%)	2321 (17.4%)	593 (8.0%)	420 (10.5%)	1120 (8.9%)	1503 (5.3%)	86 (8.1%)	498 (8.3%)	496 (7.1%)
1990s	83 (8.0%)	1286 (9.6%)	391 (5.3%)	0	0	0	16 (1.5%)	161 (2.7%)	197 (2.8%)
Pre-pandemic psychiatric diagnoses									
All diagnoses	96 (9.3%)	874 (6.6%)	517 (7.0%)	916 (22.9%)	1781 (14.2%)	4370 (15.3%)	386 (36.3%)	1487 (24.9%)	1966 (28.0%)
SCZ	<5	5 (0.0%)	6 (0.1%)	<5	<5	5 (0.0%)	<5	<5	12 (0.2%)
MDD	11 (1.1%)	157 (1.2%)	100 (1.4%)	385 (9.6%)	689 (5.5%)	1830 (6.4%)	272 (25.6%)	1020 (17.1%)	1366 (19.5%)
BPD	<5	8 (0.1%)	5 (0.1%)	46 (1.2%)	78 (0.6%)	203 (0.7%)	16 (1.5%)	56 (0.9%)	83 (1.2%)
ADHD	0	6 (0.0%)	5 (0.1%)	57 (1.4%)	58 (0.5%)	219 (0.8%)	59 (5.6%)	163 (2.7%)	250 (3.6%)

**Fig. 1:**
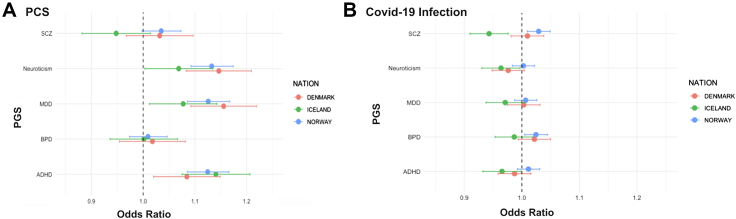
(A) Associations between standardised polygenic scores (PGS) for schizophrenia (SCZ), neuroticism, major depressive disorder (MDD), bipolar disorder (BPD), and attention-deficit/hyperactivity disorder (ADHD) and post-COVID syndrome (PCS) among individuals with confirmed COVID-19. (B) Forest plot showing associations between the same PGS and COVID-19 infection status. Odds ratios (ORs) are presented per standard deviation increase in PGS with 95% confidence intervals (CIs).

PGS's for Neuroticism, MDD and ADHD were associated with increased risk of PCS across all three cohorts (Fig. 1A). Across all three national cohorts, higher PGS for neuroticism, MDD and ADHD were consistently associated with increased odds of PCS. In the Norwegian cohort, odds ratios (ORs) of 1.13 for neuroticism (95% CI: 1.09–1.17), 1.12 for ADHD (1.09–1.17) and 1.13 for MDD (1.09–1.17) were observed. Similar findings were seen in Denmark, where neuroticism (OR = 1.13, 95% CI: 1.07–1.20), ADHD (OR = 1.08, 1.02–1.15) and MDD (OR = 1.16, 1.09–1.22) again showed positive associations with PCS. In Iceland, all three traits displayed consistent effects, with neuroticism (OR = 1.07, 95% CI: 1.00–1.13), ADHD (OR = 1.14, 1.08–1.21) and MDD (OR = 1.08, 1.01–1.14) predicting higher PCS risk (Fig. 1A). When all 15 tests were corrected jointly using FDR, the associations for ADHD and MDD remained significant in all three cohorts (e.g., ADHD FDR q-values: 8.0 × 10^−10^ in Norway, 2.9 × 10^−2^ in Denmark, 2.2 × 10^−4^ in Iceland; MDD q-values: 8.0 × 10^−10^, 3.0 × 10^−5^, 4.6 × 10^−2^, respectively). Neuroticism remained significant in Norway (q = 1.8 × 10^−10^) and Denmark (q = 2.2 × 10^−4^), but not in Iceland (q = 0.077). PGS for bipolar disorder and schizophrenia showed no FDR-corrected evidence of association with PCS in any cohort.

**Fig. 2:**
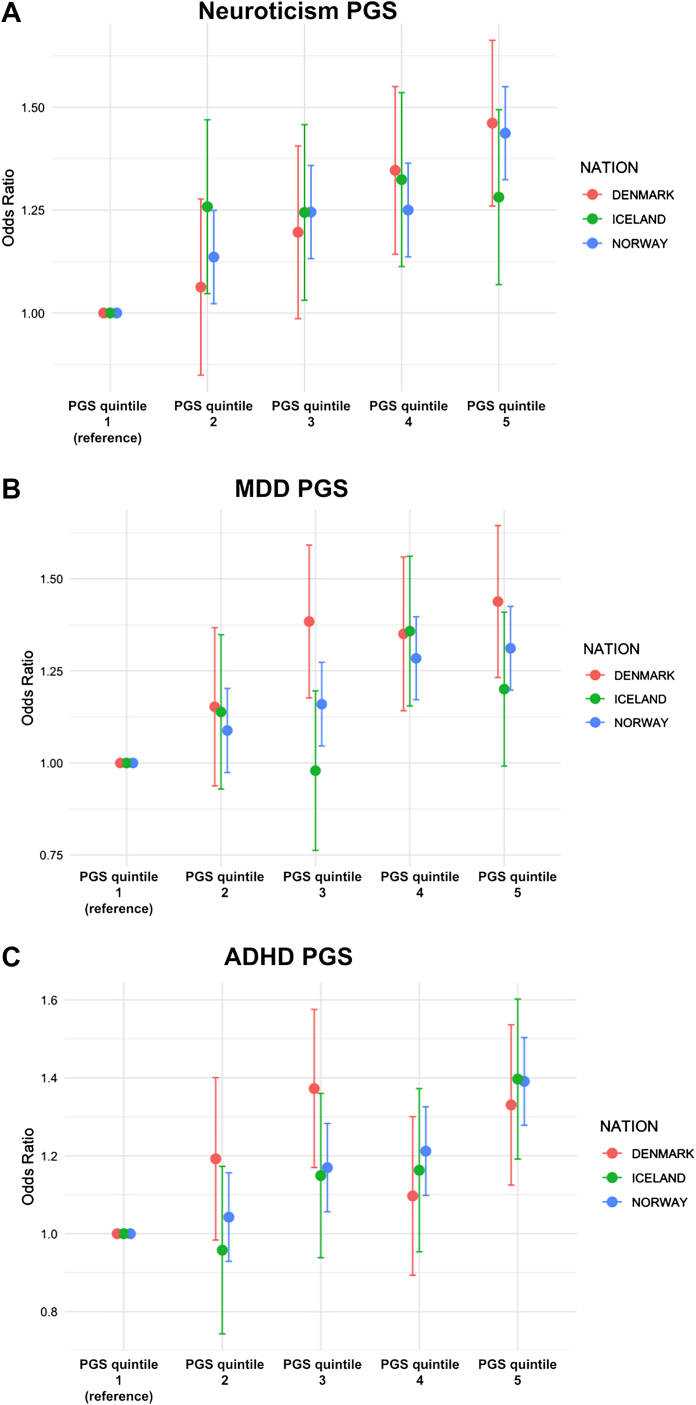
Association between psychiatric polygenic scores and post-COVID syndrome stratified by quintile. Associations between polygenic scores (PGS) for (A) neuroticism, (B) major depressive disorder (MDD), and (C) attention-deficit/hyperactivity disorder (ADHD) and post-COVID syndrome (PCS), stratified by PGS quintiles. Odds ratios (ORs) for PCS are shown for each quintile relative to the lowest quintile (reference group) within the Danish, Norwegian, and Icelandic cohorts. Odds ratios are presented with 95% confidence intervals (CIs).

Across all cohorts, higher genetic liability for neuroticism, MDD and ADHD was associated with progressively greater odds of PCS. For neuroticism, individuals in the top quintile showed odds ratios of 1.44 (95% CI: 1.32–1.55) in Norway, 1.46 (95% CI: 1.26–1.66) in Denmark, and 1.28 (95% CI: 1.07–1.49) in Iceland relative to the lowest quintile. MDD PGS showed a similar pattern, with top-quintile ORs of 1.31 (95% CI: 1.20–1.43) in Norway, 1.44 (95% CI: 1.23–1.64) in Denmark, and 1.20 (95% CI: 0.99–1.41) in Iceland. For ADHD PGS, the highest quintile was associated with 1.39 (95% CI: 1.28–1.50) in Norway, 1.33 (95% CI: 1.12–1.54) in Denmark, and 1.40 (95% CI: 1.19–1.60) in Iceland (Fig. 2).

In models assessing genetic liability for COVID-19 infection, only bipolar disorder (BPD) and schizophrenia (SCZ) PGS showed statistically significant associations and these were confined to the Norwegian cohort. In Norway, BPD PGS was associated with a modest increase in infection risk (OR = 1.02, 95% CI: 1.00–1.04), as was SCZ PGS (OR = 1.03, 95% CI: 1.01–1.05). No psychiatric PGS was associated with COVID-19 infection in the Danish cohort, and in the Icelandic cohort several point estimates were below unity, with SCZ PGS showing a small protective association (OR = 0.94, 95% CI: 0.91–0.98). Across cohorts, these COVID-19 associations were weaker than those observed for PCS (Fig. 1B).

When interaction terms were added to logistic regression models to ascertain if there were significant increases of PGS association for specific age groups, sex or occurrence of pre-pandemic psychiatric disorders no significant interactions were observed.

In a subset of the Danish cohort with available NEO Five-Factor Inventory (NEO-FFI) personality data (n = 8129), we evaluated whether measured Neuroticism accounted for the observed genetic associations. After adjusting for observed Neuroticism scores, the associations for neuroticism-PGS (adjusted OR = 1.14, 95% CI: 1.05–1.23, p = 2.2 × 10^−3^), MDD-PGS (adjusted OR = 1.11, 95% CI: 1.02–1.20, p = 1.5 × 10^−2^), and ADHD-PGS (adjusted OR = 1.13, 95% CI: 1.04–1.23, p = 3.4 × 10^−3^) remained highly similar in magnitude to the unadjusted models.

**Fig. 3:**
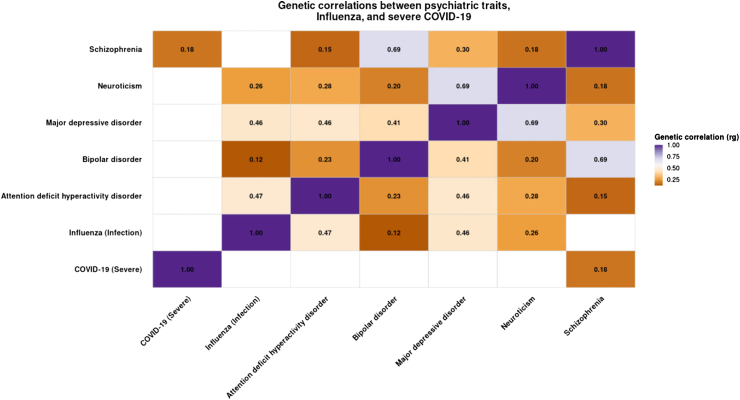
Heatmap of pairwise genetic correlations estimated using LD score regression. Pairwise genetic correlations (rg) estimated using LD score regression (LDSC) between psychiatric traits—attention-deficit/hyperactivity disorder (ADHD), major depressive disorder (MDD), bipolar disorder (BPD), schizophrenia (SCZ), and neuroticism—and two infectious disease phenotypes (influenza infection and severe COVID-19). Coloured tiles represent the magnitude and direction of the genetic correlation, with warmer colours indicating stronger positive correlations. Non-significant correlations (p ≥ 0.05) are shown as blank (white) cells. Numeric rg values are displayed for statistically significant associations only.

Genetic correlation analysis using LDSC (Fig. 3) revealed the expected pattern of substantial shared architecture among psychiatric and behavioural phenotypes, including strong correlations between bipolar disorder and schizophrenia (rg = 0.69), major depressive disorder and neuroticism (rg = 0.69), ADHD and major depression (rg = 0.46), ADHD and neuroticism (rg = 0.28), and bipolar disorder and major depression (rg = 0.41). Introducing infectious disease traits showed a divergent pattern: influenza infection demonstrated broad overlap with psychiatric liability, with significant correlations observed with ADHD (rg = 0.47), major depressive disorder (rg = 0.46), neuroticism (rg = 0.26), and bipolar disorder (rg = 0.12), whereas correlations with schizophrenia were negligible. In contrast, severe COVID-19 exhibited minimal psychiatric genetic overlap, with small and non-significant correlations with ADHD (rg = 0.03), major depressive disorder (rg = 0.14), neuroticism (rg = 0.15), and influenza (rg = 0.22); the only notable association was a modest but statistically significant correlation with schizophrenia (rg = 0.18). When summary statistics from a PGS GWAS were added only non significant correlations were found with very large confidence intervals (Supplementary Figure S1).

We examined associations between our panel of Psychiatric//behavioural PGS's and 36 inflammatory and immune-related protein markers measured on the MESOSCALE platform in 8333 Danish blood donors. ADHD PGS was the only PGS that yielded associations surviving both global and within-assay FDR correction (Table 2). Three markers showed robust ADHD PGS effects: ICAM-1 (global FDR = 4.49 × 10^−5^), CCL22 (global FDR = 3.28 × 10^−4^), and IL-1RA (global FDR = 1.10 × 10^−2^). To provide a more interpretable effect-size measure, we compared biomarker levels between the top 5% and bottom 5% of the ADHD PGS distribution. After covariate adjustment, individuals in the top 5% had 6.8% higher ICAM-1 (95% CI: 3.2%–10.4%), 6.4% higher CCL22 (95% CI: 2.1%–10.7%), and 13.7% higher IL-1RA (95% CI: 4.0%–23.5%) compared with those in the bottom 5%. To assess robustness, we repeated the analysis using non-imputed protein concentrations and observed very similar effect estimates, with all three ADHD PRS associations remaining statistically significant (ICAM-1: p = 1.47 × 10^−7^; CCL22: p = 1.49 × 10^−6^; IL-1RA: p = 1.26 × 10^−4^).

**Table 2:** Top 10 associations between psychiatric polygenic scores and circulating inflammatory protein markers.

Protein marker	PGS	p value	Global FDR	Assay FDR
ICAM-1	ADHD	1.83 × 10^−8^	4.49 × 10^−5^	1.59 × 10^−7^
CCL22	ADHD	2.64 × 10^−6^	3.28 × 10^−4^	1.32 × 10^−5^
IL-1RA	ADHD	1.35 × 10^−4^	1.10 × 10^−2^	6.76 × 10^−4^
VEGF-C	ADHD	7.41 × 10^−3^	3.11 × 10^−1^	3.71 × 10^−2^
IL-17A/F	SCZ	7.37 × 10^−3^	3.11 × 10^−1^	3.86 × 10^−2^
IL-5	Neuroticism	8.87 × 10^−3^	3.11 × 10^−1^	4.46 × 10^−2^
IL-1RA	BPD	7.66 × 10^−3^	3.11 × 10^−1^	1.92 × 10^−2^
CCL11	ADHD	1.67 × 10^−2^	4.70 × 10^−1^	8.34 × 10^−2^
IL-17A/F	BPD	1.85 × 10^−2^	4.96 × 10^−1^	4.31 × 10^−2^
IL-1B	ADHD	2.23 × 10^−2^	5.47 × 10^−1^	1.17 × 10^−1^

Protein markers showing nominal associations (p < 0.05) with at least one psychiatric polygenic score (PGS) are presented. Columns display raw p values, global false discovery rate (FDR)-adjusted p values across all tests, and assay-specific FDR-adjusted p values.

## Discussion

In this multinational study of 80,726 individuals from three population-based cohorts in Denmark, Norway and Iceland, we identified consistent associations between higher PGS for neuroticism, MDD and ADHD and increased risk of PCS. No associations were detected between PGS for SCZ or bipolar disorder BPD and PCS.

The traits showing associations with PCS; neuroticism, MDD, and ADHD all share symptom domains that strongly overlap with PCS criteria, including fatigue,^40^ concentration difficulties,^40^^,^^41^ cognitive inefficiency,^42^ sleep disturbance,^42^^,^^43^ and somatic distress.^44^ These ‘symptom-overlap traits’ provide a parsimonious explanation for why their PGSs, but not those for SCZ or BPD, predict PCS. The diagnostic and phenotypic features that define neuroticism, MDD, and ADHD map directly onto the symptom domains used to classify PCS in large-scale epidemiological studies.^45^ Individuals with higher genetic liability for these traits may therefore be more likely to experience, notice, or report symptoms that fall within the PCS construct, even in the absence of formal psychiatric diagnoses.

Conversely, SCZ and BPD involve hallmark symptoms such as hallucinations, delusions, and manic episodes, which bear no resemblance to PCS criteria.^46^^,^^47^ This mismatch in symptom domains offers a coherent explanation for the null associations observed for SCZ and BPD PGS, despite the immunologic abnormalities known in these disorders.^48^ Together, the selective pattern strongly suggests that shared symptom-related liability rather than a general psychiatric or immunologic liability underpins the associations with PCS.

Importantly, although PCS was assessed as symptoms attributed by participants to prior SARS-CoV-2 infection, our data cannot fully disentangle new or persistent post-viral sequelae from the expression or amplification of pre-existing somatic and cognitive symptom liability. Because PCS is defined through self-reported symptoms that overlap with affective and attentional traits, individuals with higher genetic liability for neuroticism, MDD, or ADHD may be more likely to experience or report symptoms within these domains following infection.

Associations for neuroticism PGS, MDD PGS, and ADHD PGS remained highly similar in magnitude after adjusting for observed neuroticism, indicating that the genetic associations are not solely proxies for measured neuroticism. Instead, they may reflect broader psychiatric liability, including subclinical or unmeasured affective and cognitive symptoms that are not fully captured by NEO-FFI scale items.

LDSC-based genetic correlations further support a non-specific interpretation. The substantial genetic correlations observed between MDD, ADHD and influenza susceptibility suggest that the identified genetic liability may reflect a broader predisposition to infection susceptibility or general illness-related symptom reporting, rather than vulnerability specific to long-term SARS-CoV-2 sequelae. These findings reinforce that the PGS–PCS associations are consistent with reflecting shared liability structures rather than virus-specific mechanisms.

Associations between our panel of PGS and inflammatory proteins were observed consistently only for ADHD-PGS, which reduces the likelihood that inflammation represents a shared pathway explaining the selective PGS–PCS associations. These results should be viewed as trait-specific biological correlates rather than mechanistic evidence for PCS.

Our study is strengthened by its large sample size, prospective design, and replication across three independent national cohorts. Genetic liability was measured prior to infection and PCS onset, reducing concerns about reverse causation. Harmonised measures of PCS across countries allowed direct comparison of findings.

Important limitations must be acknowledged. PCS remains a heterogeneous, self-reported construct, and our study cannot distinguish new onset post-viral symptoms from persistence or amplification of pre-existing symptom liability. We lacked harmonised pre-pandemic measures of psychiatric symptom severity and could therefore not test mediation or symptom-trajectory models. Similarly, our contextual analyses do not permit inference about biological pathways or causal mechanisms. Associated PGS's explained a small proportion of PCS variance, consistent with low reported SNP-heritability estimates for PCS (between 2 and 15%).^49^ These constraints mean that genetic profiling is unlikely to be clinically predictive at the individual level. Given the low reported SNP-heritability of PCS and the heterogeneity of the phenotype, we did not pursue Mendelian randomisation in the present study. Approaches such as MR or related causal inference models may be informative in future investigations with more precisely defined outcomes. However, genetic profiles reinforce the rationale for the approach used in this study, where patterns of genetic liability are employed not for prediction, but to generate insight into psychiatric and symptom-related features of PCS.

In summary, genetic liability for neuroticism, MDD, and ADHD but not schizophrenia or bipolar disorder is associated with PCS across three Nordic cohorts. The observed pattern of associations, combined with robustness to adjustment for measured neuroticism, suggests that these associations likely reflect shared symptom domains or broader underlying psychiatric liability, rather than unified immunologic or behavioural mechanisms. The present data do not allow differentiation between true post-viral sequelae and the expression or persistence of pre-existing symptom liability. Future studies with detailed pre-infection symptom assessments and longitudinal designs will be essential for determining the extent to which psychiatric liability contributes to PCS and clarifying the processes through which psychiatric liability relates to PCS.

## Contributors

LANC, LQ, and OBVP contributed to the conceptualisation of the research. LQ, IHC and IM analyzed the data. LQ drafted the manuscript and had responsibility for manuscript submission. LANC and OBVP provided close supervision to LQ and offered guidance on data analyses and data interpretation. All the authors critically reviewed the draft manuscript and approved the final version. LQ, LANC, ICC and IM have access to the data. LANC and IHC verified the data for this study.

## Data sharing statement

Person-level data from DBDS needed to reproduce this study cannot be made publicly available due to confidentiality legislation. Meta-data and programs are available from the authors upon reasonable request and with permission of the DBDS steering committee, the Ethical Committee, and the Danish Data Protection Agency. Enquiries about legal possibilities for accessing these data within DBDS, scripts/codes and further information should be addressed to the corresponding author.

## Declaration of interests

O.A. reports grants from KG Jebsen Stiftelsen, Novo Nordisk Fonden, and the NIH to his institution. He has received consulting fees from Cortechs. ai, Ledidi, and Bristol Myers Squibb (BMS), and honoraria for lectures and presentations from Lundbeck, BMS, Janssen, Eli Lilly, Otsuka, and Sunovion. He serves on the scientific advisory board of Ledidi and holds stock or stock options in Precision-Health.ai and Ledidi.

O.B.V.P. is Chairman of the Zealand Region Committee on Health Research Ethics, Denmark.

S.B. reports stock or stock option holdings in Hobe Therapeutics, Novo Nordisk A/S, Lundbeck A/S, and Eli Lilly Inc.

C.E. has received unrestricted research grants from Novo Nordisk, administered by Aarhus University, and reports no personal fees.

L.T. reports receiving payments for expert witness work in court cases related to suspected vaccine injuries.

All other authors declare no competing interests.

## Appendix A Supplementary data

Supplementary Material

Researchers in DBDS Consortium group
