# Supplementary material for: The role of genetic liability for psychiatric disorders and personality traits in post covid syndrome: data from three Nordic population cohorts
Source: eClinicalMedicine. 2026 May 7;95:103928. doi: 10.1016/j.eclinm.2026.103928 (PMC13185856; doi:10.1016/j.eclinm.2026.103928)
Supplement: Supplementary Material [file mmc1.docx]

**Supplementary Methods and Results**

**Detailed Description of genetic data duration in study cohorts**

**DBDS (Denmark)**

In DBDS, DNA from whole blood was genotyped at deCODE Genetics, using the Illumina Global Screening Array chips and long range phased using Eagle2 [^1^](https://paperpile.com/c/ni4mu3/WA7r). QC was performed prior to imputation and included removal of sample duplicates, genotype rates lower than 0.98, and ancestry outliers (less than 90) and EIGENSOFT (v 6.0.1) [^2^](https://paperpile.com/c/ni4mu3/D560). Only variants with a genotype rate higher than 0.95, a MAF > 0.01, and a p-value < 10^(-6) for HWE were retained for downstream imputation. In addition, LD pruning using a window size of 100 markers shifted by 25 markers, removed half of every variant-pair with a genotypic r^2 > 0.1. The LD pruned markers were used to calculate heterozygosity, as well as identity by state and sex. Finally, samples with outlying heterozygosity (> 5 SD from the median), a sample from each pair of samples with an IBS>0.9, samples where reported sex did not match sex determined by genotype, and all A/T and C/G markers were removed. Imputation of non-genotyped SNPs was then performed at deCODE genetics, using an inhouse workflow [^3^](https://paperpile.com/c/ni4mu3/8F7y), where graphtyper formed the reference panel. The reference panel at deCODE Genetics, Iceland included a total of approximately 25,000 individuals of North-Western European ancestry, with around 8,500 Danish individuals sequenced as part of other studies.

**MoBa (Norway)**

Genotyping of participants in MoBa has been conducted through multiple research projects, spanning several years. Different genotyping centres and arrays have been used with varying selection criteria; altogether 238,001 samples were genotyped in 24 genotyping batches. In total, 207,569 unique individuals and 6,981,748 autosomal SNPs passed the MoBaPsychGen pipeline. For further details about selection criteria and genotyping of MoBa see the cohort description [^4^](https://paperpile.com/c/ni4mu3/jyvu).

**C-19 Resilience (Iceland)**

In Iceland WGS data from 63,460 Icelanders participating in various disease projects at deCODE genetics was used as a reference panel. The samples were sequenced using standard TruSeq (Illumina) methodology to an average genome-wide coverage of 40×. SNPs and insertions and deletions (InDels) were identified, and their genotypes were called using joint calling with Graphtyper . Identified high quality variants [^5^](https://paperpile.com/c/ni4mu3/1OkV) were imputed into 173,025 chip-typed individuals using Illumina SNP arrays, and the chip-typed individuals were long-range phased [^6^](https://paperpile.com/c/ni4mu3/Rm1J).

**Supplementary Table 1 - Breakdown of GWAS summary statistics used in this study**

| **Trait** | **Discovery GWAS** | **Publication link** | **PMID** |
| --- | --- | --- | --- |
| SCZ | Trubetskoy et al., 2022 | 10.1038/s415 86-022-04434- 5 | 35396580 |
| Neuroticism | Nagel et al., 2018 | https://doi.org /10.1038/s415 88-018-0151-7 | 29942085 |
| MDD | Howard et al., 2019 | https://doi.org /10.1038/s415 93-018-0326-7 | 30718901 |
| BPD | Mullins et al., 2021 | https://doi.org /10.1038/s415 | 34002096 |
| ADHD | Demontis et al., 2019 | https://doi.org /10.1038/s415 88-018-0269-7 | 30478444 |
| Influenza | Kosmicki et al, 2024 | <https://doi.org/10.1038/s41588-024-01844-1> | 39103650 |
| COVID-19 severity | Degenhardt et al, 2022 | 10.1093/hmg/ddac158 | 35848942 |

**Supplementary Figure 1 - Forest plot of LDSC associations and PGS panel**

**
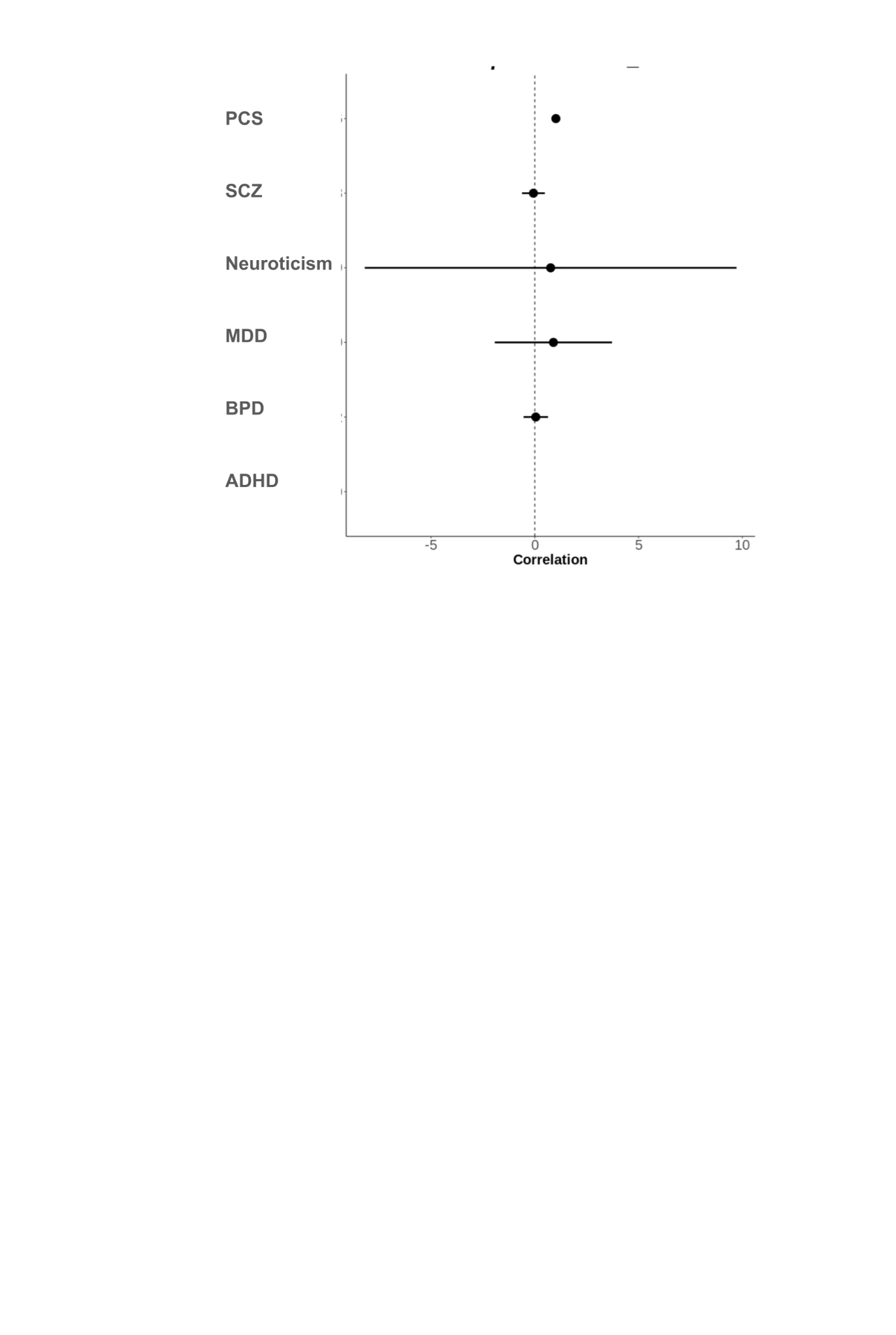
**

**Bibliography**

**1.** [**Loh, P.-R., Danecek, P., Palamara, P.F., Fuchsberger, C., A Reshef, Y., K Finucane, H., Schoenherr, S., Forer, L., McCarthy, S., Abecasis, G.R., et al. (2016). Reference-based phasing using the Haplotype Reference Consortium panel. Nat Genet *48*, 1443–1448.**](http://paperpile.com/b/ni4mu3/WA7r)

**2.** [**Patterson, N., Price, A.L., and Reich, D. (2006). Population structure and eigenanalysis. PLoS Genet *2*, e190.**](http://paperpile.com/b/ni4mu3/D560)

**3.** [**Gudbjartsson, D.F., Helgason, H., Gudjonsson, S.A., Zink, F., Oddson, A., Gylfason, A., Besenbacher, S., Magnusson, G., Halldorsson, B.V., Hjartarson, E., et al. (2015). Large-scale whole-genome sequencing of the Icelandic population. Nat Genet *47*, 435–444.**](http://paperpile.com/b/ni4mu3/8F7y)

**4.** [**Brandlistuen, R.E., Kristjansson, D., Alsaker, E., Valen, R., Birkeland, E., Røyrvik, E.C., Page, C.M., Aamelfot, M., Vangbæk, S., Ask, H., et al. (2025). Cohort Profile Update: The Norwegian Mother, Father and Child Cohort (MoBa). Int J Epidemiol *54*. https://doi.org/**](http://paperpile.com/b/ni4mu3/jyvu)[**10.1093/ije/dyaf139**](http://dx.doi.org/10.1093/ije/dyaf139)[**.**](http://paperpile.com/b/ni4mu3/jyvu)

**5.** [**Eggertsson, H.P., Jonsson, H., Kristmundsdottir, S., Hjartarson, E., Kehr, B., Masson, G., Zink, F., Hjorleifsson, K.E., Jonasdottir, A., Jonasdottir, A., et al. (2017). Graphtyper enables population-scale genotyping using pangenome graphs. Nat Genet *49*, 1654–1660.**](http://paperpile.com/b/ni4mu3/1OkV)

**6.** [**Kong, A., Masson, G., Frigge, M.L., Gylfason, A., Zusmanovich, P., Thorleifsson, G., Olason, P.I., Ingason, A., Steinberg, S., Rafnar, T., et al. (2008). Detection of sharing by descent, long-range phasing and haplotype imputation. Nat Genet *40*, 1068–1075.**](http://paperpile.com/b/ni4mu3/Rm1J)
