# Supplementary material for: The role of genetic liability for psychiatric disorders and personality traits in post covid syndrome: data from three Nordic population cohorts
Source: eClinicalMedicine. 2026 May 7;95:103928. doi: 10.1016/j.eclinm.2026.103928 (PMC13185856; doi:10.1016/j.eclinm.2026.103928)
Supplement: Researchers in DBDS Consortium group [file mmc2.pdf]

DBDS Consortium

Researchers in DBDS Consortium

Name	Afilliation
Jakob Hjorth von Stemann	Region Høvestaden, Danmark
Nanna Brøns	Region Høvestaden, Danmark
Josephine Gladov	Region Midtjylland, Danmark
Lotte Hindhede	Region Midtjylland, Danmark
Maiken Astrup Madsen	Region Midtjylland, Danmark
Lea Arregui Nordahl Christoffersen	Region Høvestaden, Danmark
Liam Quinn	Region Zealand, Danmark
Jacob Træholt	Region Høvestaden, Danmark
Bertram Kjerulff	Region Midtjylland, Danmark
Jens Kjærgaard Boldsen	Region Midtjylland, Danmark
Johan Skov Bundgaard	Region Høvestaden, Danmark
Line Hjorth Stjernholm Nielsen	Region Midtjylland, Danmark
Mette Skou Bentsen	Region Høvestaden, Danmark
Khoa Manh Dinh	Region Høvestaden, Danmark
Joseph Dowsett	Region Høvestaden, Danmark
Maria Didriksen	Region Zealand, Danmark
Michael Schwinn	Region Høvestaden, Danmark
Lisette kogelman	Region Høvestaden, Danmark
Anne Grosen	Region Midtjylland, Danmark
Tanya Techlo	Region Høvestaden, Danmark
Christina MIkkelsen	Region Høvestaden, Danmark
Thomas F Hansen	Region Høvestaden, Danmark
Susan Mikkelsen	Region Midtjylland, Danmark
Kathrine A Kaspersen	Region Midtjylland, Danmark
Laura Barrett Ryø	Region Midtjylland, Danmark
Rasmus Tanderup Jensen	Region Høvestaden, Danmark
Casia Nursyifa	Region Zealand, Danmark
Caroline Thue Hvilsom	Region Midtjylland, Danmark
Emil Jørsboe	Region Zealand, Danmark
